# Small amounts of ethanol attenuate folic acid stability in acidic beverages during storage

**DOI:** 10.1002/fsn3.549

**Published:** 2017-11-20

**Authors:** Kaori Kida, Muneaki Tomotake, Hiroshi Sasako, Yoshihito Matsuda, Naomi Sasaki, Norio Yamamoto

**Affiliations:** ^1^ Development Division 2 Research & Development Institute House Wellness Foods Corporation Yotsukaido Chiba Japan; ^2^ Central Research & Development Institute House Foods Group Inc. Yotsukaido Chiba Japan; ^3^ R&D Planning Division Research & Development Institute House Wellness Foods Corporation Itami Hyogo Japan

**Keywords:** beverage, ethanol, folic acid, proton attack, stability

## Abstract

Folic acid (FA) is an essential compound involved in important biochemical processes and is used to fortify various food products. FA in fortified acidic beverages decomposes during storage due to H^+^ attack. FA stability in acidic beverages is a serious problem as food fortification should guarantee labeled FA concentrations until the expiry date. In this study, we investigated the influence of ethanol (EtOH) on FA depletion using a model acidic beverage and observed that small amounts of EtOH, derived from added flavor, promoted FA depletion. FA depletion was promoted by only small amounts of EtOH, but not by acetonitrile. This suggested that FA decomposition might be accelerated by EtOH, which surrounds FA molecules in solution due to selective solvation. In the development of FA‐fortified beverages, EtOH content should be decreased or removed altogether, to prevent accelerating FA decomposition.

## 1 INTRODUCTION

**Figure 1:**
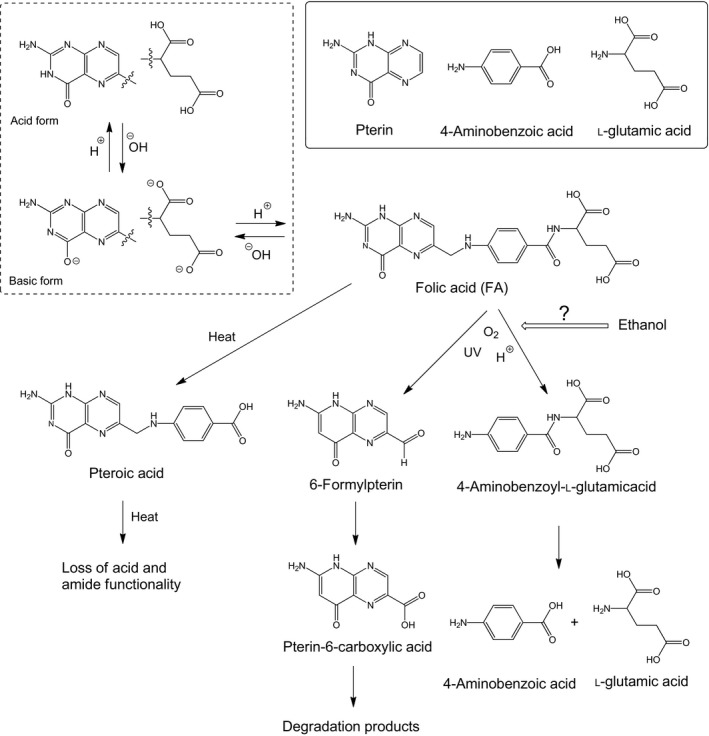
Molecular structure and degradation scheme of folic acid (Gazzali et al., 2016). Folic acid is composed of different moieties, namely pterin, 4‐aminobenzoic acid, and l‐glutamic acid (inset, top right). The pterin moiety of folic acid structure can convert between acidic and basic forms (inset, top left)

Folate is a water‐soluble B vitamin (B_9_) important in cell multiplication, the regulation of gene activity, red and white blood cell production, the renewal of skin and intestinal lining, and the synthesis of chemicals that modulate brain function (Gazzali et al., 2016). Folate deficiency classically presents as macrocytic anemia. Folate occurs naturally in foods such as legumes, leafy green vegetables, and some citrus fruits (Gazzali et al., 2016; US Preventive Services Task Force [USPSTF], 2017). Folic acid (FA; the monoglutamate form of folate, pteroyl‐l‐monoglutamic acid) is the synthetic and most stable form of folate, and is often added to dietary supplements and fortified foods. There is evidence that sufficient fortification of the diet with FA and vitamin B_12_ can reduce the risk of heart disease by decreasing total homocysteine (De‐Regil, Finkelstein, Sæterdal, Gaitán, & Peña‐Rosas, 2016). FA fortification has also been shown to reduce the risk of neural tube defects, with up to 70% estimated to be prevented by increasing FA intake during periconception (Czeizel, Dudás, Vereczkey, & Bánhidy, 2013; De‐Regil et al., 2016). The World Health Organization (WHO) recently published guidelines for optimal red blood cell folate and serum folate concentrations in women of reproductive age to prevent neural tube defects. The recommended cut‐off for preventing neural tube defects is a red blood cell folate concentration of 906 nmol·L^−1^ (<400 μg·L^−1^), which is an indicator of longer term folate status (World Health Organization [WHO], 2015). The USPSTF recommended in 2009 that all women planning or capable of pregnancy take a daily supplement containing 400–800 μg of FA (USPSTF, 2017). The molecular structure of FA can be considered as three moieties: glutamic acid (Glu), 4‐aminobenzoic acid (PABA), and pterin (Figure 1). The pterin moiety is linked to PABA by a methylene bridge, while PABA is connected to Glu by an amide bond (Gazzali et al., 2016). FA has low solubility in water and is practically insoluble at 25°C (10 mg·L^−1^). Its solubility improves in alkaline or acidic media, but FA is more stable in alkaline media (Gazzali et al., 2016). As shown Figure 1, FA degradation is caused by several factors, such as high temperature, light, low pH, oxygen, and food composition (Nguyen, Oey, Verlinde, van Loey, & Hendrickx, 2003; Fukuwatari, Fujita, & Shibata, 2009; Jastrebova, Axelsson, Strandler, & Jägerstad, 2013; Gazzali et al., 2016). Degradation occurs during storage, especially in vitamin juices, fruit juice mixtures fortified with several vitamins, which have low pH values (3.5) (Frommherz et al., 2014). Many beverages enriched with FA are adjusted to be weakly acidic for microbial control. Our company has produced a FA‐fortified bottled beverage (pH 3.5–3.7), in which FA depletion during storage was a serious problem. Food fortification should guarantee the labeled FA concentration until the expiry date, which usually entails adding larger amounts of the vitamin to compensate for losses during processing and/or storage (Frommherz et al., 2014). Proposed systems have been developed to improve FA stability in different food matrices and food processing procedures (Ruiz‐Rico et al., 2017). To improve FA stability, we have also investigated factors that promote FA depletion by other ingredients in the aforementioned beverage produced by our company, noting that the small amount of ethanol (EtOH) present in a flavoring food additive promoted FA depletion. In this study, we have confirmed that small amounts of EtOH promote FA depletion in an acidic beverage and investigated the mechanism of enhanced FA depletion in the presence of EtOH.

## 2 EXPERIMENTAL PROCEDURE

### 2.1 Materials and chemicals

FA was purchased from DSM Nutrition Japan (Tokyo, Japan). Acesulfame K and sucralose were obtained from San‐Ei Gen F.F.I. (Osaka, Japan). High‐fructose corn syrup, reduced malt sugar syrup, citric acid, and trisodium citrate were of food grade. All other reagents were of the highest grade available from commercial sources. Flavors #1, #2, and #3 were made to order by flavor companies in Japan for the beverage produced by our company.

### 2.2 Preparation of test solutions of model beverage

Model beverages were prepared from deionized water, high‐fructose corn syrup, sucrose, reduced malt sugar syrup, ascorbic acid, citric acid, and trisodium citrate among other ingredients (Table 1). All model beverages contained FA (final concentration of 3.16 mg·L^−1^) and were adjusted to pH 3.5 using 3 mol·L^−1^ NaOH and 3 mol·L^−1^ HCl after adding the test ingredient. The prepared model beverages were added into laminated retort pouches with aluminum foil (50 ml each) at room temperature and then sterilized by heating to 90°C and folding for 10 min in an incubator. After sterilization, the pouches were cooled in cold water for 30 min.

**Table 1:** Composition of model beverage base used in this study

Component	Amount per L
Folic acid	3.16 mg
Sucrose (granulated sugar)	24.5 g
High‐fructose corn syrup	11.4 g
Reduced malt sugar syrup	5.3 g
Sucralose (artificial sweetener)	37 mg
Acesulfame K (artificial sweetener)	132 mg
Sweetener formula (0.15% of thaumatin)	5 mg
Citric acid	2.0 g
Sodium citrate	2.0 g
l‐Ascorbic acid	7.7 g
Deionized water	Fill up to 1 L

### 2.3 Storage stability test

To evaluate the storage stability of FA, the retort pouches were stored in an incubator at 50°C for 6 or 13 days as an accelerated shelf‐life time test. During incubation, pouches were removed during each sampling period, frozen, and stored at −20°C until FA stability analysis.

### 2.4 Determination of folic acid

FA concentrations were determined by high‐performance liquid chromatography (HPLC). Briefly, the frozen sample in the retort pouch was thawed in tap water and the thawed solution transferred to a beaker. The sample solution was adjusted to pH 12 using 1 mol·L^−1^ NaOH and stirred for 20 min at room temperature. After stirring, the sample solution was readjusted to pH 9.5–10.5 using 1 mol/L HCl, filtered through a 0.45‐μm polytetrafluoroethylene membrane filter (Advanced Microdevices, Ambala Cantt, India), and injected into the HPLC system (ACQUITY H Class, Waters, MA, USA) using the following setup: column, XBridge C18 (5 μm, 6 × 250 mm; Waters); mobile phase, methanol/H_2_O (12:88, v/v) containing 0.3% (v/v) acetic acid, and 1.08% (w/v) 1‐octanesulfonic acid sodium salt; flow rate, 1.2 ml·min^−1^; temperature, 50°C; UV detection, 292 nm; data analysis, Empower 3 (Waters). These conditions of HPLC were originally developed because a peak of FA needs to be separated from peaks of other additives. FA concentration was calculated from the standard curve generated using standard FA solution prepared in advance.

## 3 RESULTS

### 3.1 Screening of factors enhancing folic acid depletion in model beverage

We began by screening factors that enhanced FA depletion. As shown in Table 2, flavor #3 was the most effective at depleting FA in the model beverage during storage at 50°C. As flavors often contain EtOH, the EtOH concentrations in flavors used in this examination were measured. Flavors #1, #2, and #3 contained small amounts of EtOH, giving final EtOH concentrations in the model beverage of 0.008%, 0.03%, and 0.096% (v/v), respectively. FA content decreases in acidic solutions, such as acidic beverages, over time (Frommherz et al., 2014), with the main mechanism of FA decomposition under acidic conditions being proton attack (Gazzali et al., 2016). Therefore, we expected even small amounts of EtOH to enhance FA decomposition in acidic solution.

**Table 2:** Effects of ingredients on folic acid stability in model beverage stored at 50°C

Ingredients (amount in preparation)	Concentration of folic acid, mg·L^−1^ (residual ratio, %)		
	Day 0^a^	Day 5	Day 13
Nicotinamide (70 mg·L^−1^)	2.610^b^ (82.6)^c^	2.164 (68.5)	2.056 (65.1)
Dibenzoyl thiamine hydrochloride (15.3 mg·L^−1^)	2.336 (73.9)	2.012 (63.7)	1.782 (56.4)
Pyridoxine hydrochloride (0.7 mg·L^−1^)	2.578 (81.6)	2.110 (66.8)	1.940 (61.4)
Vitamin mix formulation^d^ (1,740 mg·L^−1^)	2.556 (80.9)	1.923 (60.9)	1.872 (59.2)
Calcium pantothenate (160 mg·L^−1^)	2.662 (84.2)	2.057 (65.1)	1.918 (60.7)
Cyanocobalamin (31.6 μg·L^−1^)	2.614 (82.7)	1.886 (59.7)	1.821 (57.6)
Riboflavin (10 mg·L^−1^)	2.653 (84.0)	1.912 (60.5)	1.438 (45.5)
Biotin (0.3 mg·L^−1^)	2.592 (82.0)	2.005 (63.5)	1.763 (55.8)
Vitamin K formulation^e^ (0.4 mg·L^−1^)	2.688 (85.1)	2.176 (68.9)	1.825 (57.8)
Concentrated grapefruit juice (8.2 g·L^−1^)	2.575 (81.5)	1.945 (61.6)	1.688 (53.4)
Flavor #1 (230 mg·L^−1^)	2.452 (77.6)	1.832 (58.0)	1.719 (54.4)
Flavor #2 (380 mg·L^−1^)	2.453 (77.6)	1.414 (44.8)	1.676 (53.0)
Flavor #3 (150 mg·L^−1^)	2.094 (66.3)	**0.939 (29.7)**	**0.716 (22.7)**

a Data recorded immediately after preparation of test pseudo beverage.

b Mean value of duplicate determination (*n* = 2).

c Percentage of folic acid in preparation (3.16 mg·L^−1^).

d Emulsified formulation containing vitamins A, E, and D.

e Emulsified formulation of vitamin K.

Residual ratio is less than 40%.

### 3.2 Effect of ethanol on FA stability in acidic beverage

**Figure 2:**
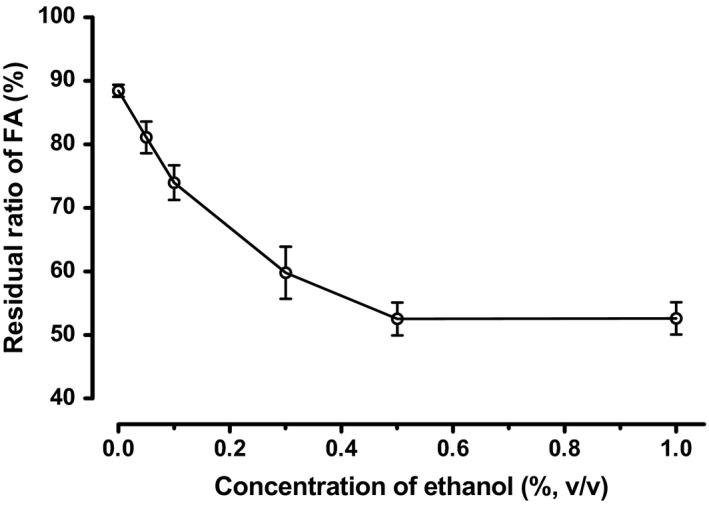
Concentration‐dependent effect of ethanol on folic acid (FA) stability during storage at 50°C for 6 days. Ethanol was added to the model beverage base (Table 1) at concentrations of 0%, 0.05%, 0.1%, 0.3%, 0.5%, and 1% (v/v). The model beverages were added into retort pouches and stored at 50°C. The residual ratio of FA is shown as the percentage of FA in the preparation (3.16 mg·L^−1^). Data are expressed as *M* ± *SD* (*n* = 5)

To confirm EtOH‐promoted FA decomposition in acidic beverages, a storage test was conducted by adding EtOH to the base model beverage. The amounts of EtOH added were 0%, 0.05%, 0.1%, 0.3%, 0.5%, and 1% (v/v). As shown in Figure 2, EtOH promoted a dose‐dependent decrease in FA content. A recent review by Gazzali et al. (2016) highlighted important factors that can affect FA decomposition, including light, heat, oxygen, pH, and concentration. However, the promotion of FA decomposition by small amounts of EtOH in acidic solution is little known, with no mechanism reported. As EtOH concentrations in the model beverage were small, it was unlikely that EtOH and FA were reacting directly, despite the amount of EtOH being larger than the amount of FA. Ethanol can form adducts by dehydration condensation, but since ethanol adducts rather hydrolyze in acidic aqueous solution, it is unlikely that ethanol will be added to FA by dehydration condensation. Akhtar, Ataullah, and Ahmad (1999) reported that the solubility of FA at pH 3.5 was 2.42 μmol·L^−1^ in evaluating photostability of FA that was added at a concentration of 50 μmol·L^−1^ in various pH of aqueous solution. In their study, the solubility of FA at pH 4.2 was 4.80 μmol·L^−1^, despite at pH 4.5 or higher, all 50 μmol was dissolved, thus FA solubility increased over 10 times between pH 4.2 and 4.5 (Akhtar et al., 1999). Since the concentration of FA added in our study was 7.16 μmol·L^−1^ (3.16 mg·L^−1^), it was thought that FA in our acidic beverage (pH 3.5) was not completely dissolved and it existed also as dispersion of nanoclusters. Therefore, the following potential reaction mechanisms were considered: (1) EtOH increases FA solubility and/or (2) EtOH increases the proton exchange rate. Both mechanisms would lead to increased FA decomposition. To verify these hypotheses, the following experiments were conducted.

### 3.3 Comparison of effects of ethanol and acetonitrile on folic acid stability

**Figure 3:**
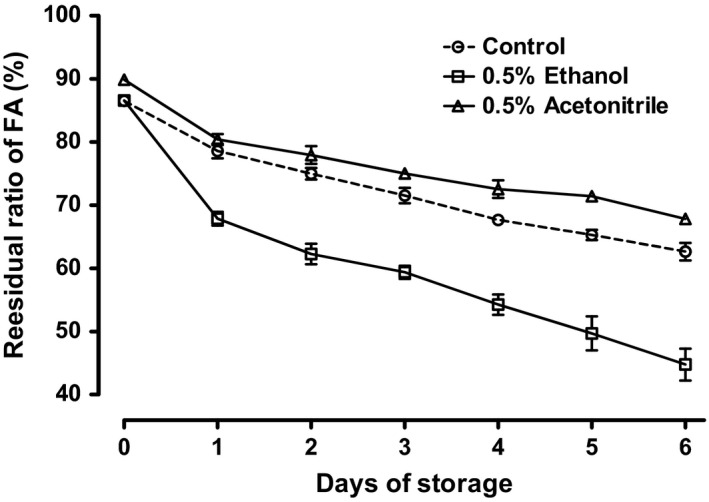
Comparison of changes in folic acid (FA) stability with the addition of ethanol and acetonitrile. Ethanol and acetonitrile were added at concentrations of 0.5% (v/v) to model beverage base solution (Table 1). Each sample was added into a retort pouch and stored at 50°C for up to 6 days. A control was prepared from base solution only. Aliquots for quantification of FA were taken from each pouch daily and FA concentrations were determined by HPLC. The residual ratio is shown as the percentage of FA in the preparation (3.16 mg·L^−1^). Data are expressed as *M* ± SD (*n* = 5)

The first and second acid dissociation constants (p*K*_a_) of FA are 2.3 (from the glutamic acid moiety) and 8.3 (from the pterin moiety). As the pterin moiety of FA molecule might exist in its nonionic form in acidic solution at pH 3.5, FA would not completely dissolve at a concentration of 3.16 mg·L^−1^. In solute molecules containing hydrophobic and hydrophilic moieties, hydrophobic hydration is disturbed by adjacent polar groups (Ishihara, Okouchi, & Uedaira, 1997). In fact, FA is not easily hydrated with an acidic aqueous solution. According to Akhtar et al. (1999), approximately half of FA added at a concentration of 3.16 mg·L^−1^ in the solution at pH 3.5 should not be dissolved and FA might form FA nanoclusters. It is known that polar organic solvents, such as EtOH, are present in high concentrations near organic molecules in aqueous solutions due to selective solvation (Onuki, Okamoto, & Araki, 2011). The PABA moiety of FA structure is considered to be particularly compatible with organic solvents because PABA itself dissolves well in polar and nonpolar organic solvents. Therefore, EtOH was assumed to gather around FA nanoclusters by selective solvation, enhancing the solubility and, consequently, the reactivity of FA. To confirm this, acetonitrile and EtOH, which have similar polarities (relative polarity: EtOH, 0.654; acetonitrile 0.460, eluent length: EtOH, 0.88; acetonitrile, 0.65), were added in 0.5% (v/v) concentration to the model beverage (pH 3.5) to confirm FA attenuation. As shown in Figure 3, EtOH‐promoted FA depletion, while acetonitrile did not. This result confirmed that increasing FA solubility through selective solvation was not a major factor in FA depletion. As proton attack has been shown to cause FA decomposition in acidic solutions (Gazzali et al., 2016), the EtOH hydroxyl residue was expected to be the main factor promoting FA degradation.

## 4 DISCUSSION

In this study, we confirmed that the presence of small amounts of EtOH (0.1–1%, v/v) promoted FA depletion in acidic beverages, while an equivalent amount of acetonitrile had no effect under the same conditions. Water is the most important constituent in beverages, while various components influence process variables, product characteristics, and stability attributes. Solvation is also referred to as hydration in the case of water and aqueous mixtures. In mixtures of water‐like and less polar fluids (including polymer solutions), solvation is preferential or selective, depending on whether a solute is hydrophilic or hydrophobic (Onuki et al., 2011). The main mechanism for FA decomposition in acidic solution is hydrolysis by proton attack (Figure 1) (Gazzali et al., 2016). The main sources of H^+^ attack are acid (citric acid in this study) and H_2_O. Therefore, it is important to understand changes in H^+^ (H_3_O^+^) characteristics in the presence of EtOH. Previously, Nose and Hojo (2006) confirmed that the proton exchange rate was higher in EtOH–H_2_O solutions containing organic acid. In contrast, they observed that sugars had no apparent strengthening effect on the hydrogen‐bonding structure in EtOH–H_2_O, and that the presence of EtOH strengthened EtOH–H_2_O hydrogen bonds (Nose & Hojo, 2006). Adding EtOH accelerated FA decomposition in a concentration‐dependent manner at concentrations up to 0.5% (v/v), with no further increase observed when the EtOH concentration was increased to 1% (v/v) (Figure 2). Therefore, the amount of EtOH surrounding the FA molecules and/or FA clusters was considered saturated at 0.5% (v/v). EtOH can gather around FA clusters by selective solvation, even at low EtOH concentrations, making the FA clusters more reactive. Therefore, we speculated that increasing the presence of EtOH around FA clusters increased the proton exchange rate, which promoted FA decomposition. Although selective solvation is relevant in diverse fields, there is still a limited understanding of this phenomenon (Onuki et al., 2011). To clearly explain this observation, further experiments are necessary.

## 5 CONCLUSIONS

FA is commonly used in food fortification to improve human health. However, FA stability in acidic beverages is a serious problem as food fortification should guarantee labeled FA concentrations until the expiry date. To prevent FA depletion, mechanistic understanding of this process is required. Previous studies have focused on various factors and proposed methods for FA stabilization, such as organic or inorganic encapsulation of FA. In this study, we observed that small amounts of EtOH, even below 0.5% (v/v), promoted FA depletion in acidic beverages. This phenomenon suggested that EtOH, which surrounds FA clusters by selective solvation, promoted FA decomposition in acidic beverages. To fully explain this phenomenon, further experiments are necessary. However, the presence of even small amounts of EtOH, such as those derived from food additives (e.g., flavors), clearly promoted FA depletion in acidic beverages. To develop FA‐fortified beverages, the EtOH content should be decreased, or preferably removed altogether.

## CONFLICT OF INTEREST

None declared.
